# MRI detection of breast cancer micrometastases with a fibronectin-targeting contrast agent

**DOI:** 10.1038/ncomms8984

**Published:** 2015-08-12

**Authors:** Zhuxian Zhou, Mohammed Qutaish, Zheng Han, Rebecca M. Schur, Yiqiao Liu, David L. Wilson, Zheng-Rong Lu

**Affiliations:** 1 Department of Biomedical Engineering, Case Western Reserve University, Cleveland, Ohio 44106, USA; 2 Center for Bionanoengineering and State Key Laboratory of Chemical Engineering, Department of Chemical and Biological Engineering, Zhejiang University, Hangzhou 310027 China; 3 Department of Radiology, Case Western Reserve University, Cleveland, Ohio 44106, USA

## Abstract

Metastasis is the primary cause of death in breast cancer patients. Early detection of high-risk breast cancer, including micrometastasis, is critical in tailoring appropriate and effective interventional therapies. Increased fibronectin expression, a hallmark of epithelial-to-mesenchymal transition, is associated with high-risk breast cancer and metastasis. We have previously developed a penta-peptide CREKA (Cys-Arg-Glu-Lys-Ala)-targeted gadolinium-based magnetic resonance imaging (MRI) contrast agent, CREKA-Tris(Gd-DOTA)_3_ (Gd-DOTA (4,7,10-tris(carboxymethyl)-1,4,7,10-tetraazacyclododecyl gadolinium), which binds to fibrin–fibronectin complexes that are abundant in the tumour microenvironment of fast-growing breast cancer. Here we assess the capability of CREKA-Tris(Gd-DOTA)_3_ to detect micrometastasis with MRI in co-registration with high-resolution fluorescence cryo-imaging in female mice bearing metastatic 4T1 breast tumours. We find that CREKA-Tris(Gd-DOTA)_3_ provides robust contrast enhancement in the metastatic tumours and enables the detection of micrometastases of size <0.5 mm, extending the detection limit of the current clinical imaging modalities. These results demonstrate that molecular MRI with CREKA-Tris(Gd-DOTA)_3_ may facilitate early detection of high-risk breast cancer and micrometastasis in the clinic.

## Abstract (web summary)

Increased fibronectin expression is associated with high-risk breast cancer and metastasis. Here, the authors develop a penta-peptide CREKA-targeted MRI contrast agent that binds to fibronectin and show its potential for early detection of breast cancer and micrometastasis.

## Graphical abstract

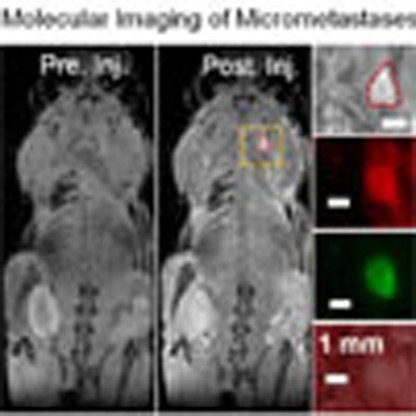

Breast cancer has a high rate of metastasis; one-third of the patients diagnosed with breast cancer eventually develop metastases in distant organs, with an increased risk of mortality^1^. Breast cancer primarily metastasizes to the bone, lung, liver, lymph nodes and brain^2^,^3^. Breast cancer metastasis can occur years after apparently successful treatment, underscoring the importance of efficient clinical management of the disease, including prompt treatment response and monitoring for possible relapse. Early and accurate detection and differential diagnosis of breast cancer with metastatic potential and micrometastasis (<2 mm) may facilitate the design of more effective and time-sensitive patient-specific therapies^4^,^5^. Current clinical imaging modalities demonstrate limited potential in the detection and differential diagnosis of small high-risk breast cancer (<2 mm) and micrometastasis. Magnetic resonance imaging (MRI) is a powerful technique for high-resolution visualization of the anatomic structure and function of soft tissues, including tumours^6^. Small molecular Gd(III) chelates are routinely used for clinical cancer imaging to enhance image contrast by shortening the relaxation times of the surrounding water protons^7^. However, these chelates are non-specific contrast agents and cannot differentiate tumour aggressiveness or provide efficient detectable contrast in small tumours and micrometastases. Consequently, molecular imaging using a biomarker that is specifically associated with tumour aggressiveness and metastasis is an effective approach towards the early detection and differential diagnosis of high-risk breast cancer.

Tumour microenvironment plays an essential role in tumour progression and metastasis^8^,^9^. A major component of the microenvironment is the extracellular matrix (ECM), which is composed of distinct components including collagen, proteoglycans, laminins and fibronectin. Compared with normal tissue, the tumour ECM is highly deregulated, with fundamentally different composition, architecture, biochemistry and physical properties^10^. Fibronectin is abundantly expressed in several types of malignant tumours and is associated with an invasive and metastatic phenotype^11^,^12^,^13^. Tumour fibronectin has been used as a biomarker to develop antibody-targeted vehicles for specific and effective delivery of imaging agents and therapeutic drugs to metastatic sites^14^,^15^,^16^. Changes in the production and organization of fibronectin in the ECM contribute to the ‘pre-metastatic niche', which dictates the pattern of metastatic spread^17^,^18^. The expression of fibronectin is highly upregulated by transforming growth factor-beta (TGF-β) during epithelial-to-mesenchymal transition (EMT), and is a hallmark of EMT^19^,^20^. According to The Cancer Genome Atlas (TCGA)-National Cancer Institute (NCI), invasive ductal breast carcinoma and breast carcinoma exhibit a six to sevenfold increase in the fibronectin expression, compared with normal breast (http://cancergenome.nih.gov). Increased fibronectin expression in the pre-metastatic niche facilitates the adhesion of bone marrow-derived cells, which promote tumour progression and metastasis^17^,^21^. Since the fibronectin content in tumours is associated with their growth and angiogenesis^22^, determination of the fibronectin content can serve as a prognostic biomarker for breast cancer^23^. The deposition of fibronectin into the tumour ECM, followed by the formation of fibrin–fibronectin complexes has been shown to facilitate tumour proliferation, angiogenesis and metastasis^11^. Thus, the highly expressed fibronectin and its complex with other matrix proteins such as fibrin are attractive biomarkers in molecular imaging for the early detection and differential diagnosis of high-risk breast cancer and micrometastasis.

To enhance MRI sensitivity for cancer molecular imaging, the selection of appropriate molecular biomarkers and rational design and development of contrast agents are imperative^24^,^25^,^26^,^27^,^28^,^29^,^30^. We have developed a penta-peptide CREKA-targeted MRI contrast agent, CREKA-Tris(Gd-DOTA)_3_ (Gd-DOTA (4,7,10-tris(carboxymethyl)-1,4,7,10-tetraazacyclododecyl gadolinium)), for cancer molecular imaging with contrast-enhanced MRI^28^. The CREKA peptide specifically binds to fibrin–fibronectin complexes in tumour ECM, with negligible binding in normal tissues^31^,^32^. Consequently, the CREKA-targeted contrast agent also binds to the abundant fibrin–fibronectin complexes in the tumour ECM, producing robust and prolonged tumour contrast enhancement, as compared with non-targeted control, in a primary mouse tumour model^28^. We hypothesize that the upregulated expression of fibronectin and fibrin–fibronectin complexes in the ECM of high-risk and metastatic breast cancers can be used as a biomarker to facilitate the detection of small high-risk breast cancer and micrometastases, using the CREKA-targeted contrast agent for non-invasive high-resolution molecular MRI (Fig. 1). Here we assess the potential of CREKA-Tris(Gd-DOTA)_3_ in high-resolution molecular MRI of small aggressive breast cancer and micrometastases in mouse metastatic breast cancer models. The effectiveness of molecular MRI is further validated by a three-dimensional (3D) fluorescence cryo-imaging technique with CREKA-Cy5, which provides 3D whole-body images of metastases at micron resolution^33^,^34^.

## Results

### Fibronectin expression in metastatic tumours and normal tissues

EMT is a key step in the initiation of invasion and metastases of high-risk breast cancer. TGF-β is a major inducer of EMT and promotes ECM production^35^,^36^. The treatment of 4T1 breast cancer cells with TGF-β resulted in a fibroblast-like mesenchymal phenotype (Fig. 2a), accompanied by the appearance of the features of EMT, including upregulation of the mesenchymal marker fibronectin and downregulation of the epithelial marker E-cadherin, as determined by quantitative PCR (Fig. 2b). Fibronectin expression in metastatic tumours and normal tissues was determined by western blot in both primary and metastatic breast cancers induced by inoculating 4T1–GFP-Luc2 cancer cells in the mammary fat pad of the Balb/c mouse tumour model. As shown in Fig. 2c,d (Supplementary Fig. 1), both the primary and metastatic tumours isolated from different tissues exhibit increased levels of fibronectin. The fibronectin expression in the metastatic tumours was relatively higher than that in the primary tumours. In contrast, fibronectin expression in the normal lung and brain tissues was much lower than that in the tumours, except the liver, where plasma fibronectin is normally produced.

### CREKA binds to fibrin–fibronectin complexes in metastases

**Figure 1:**
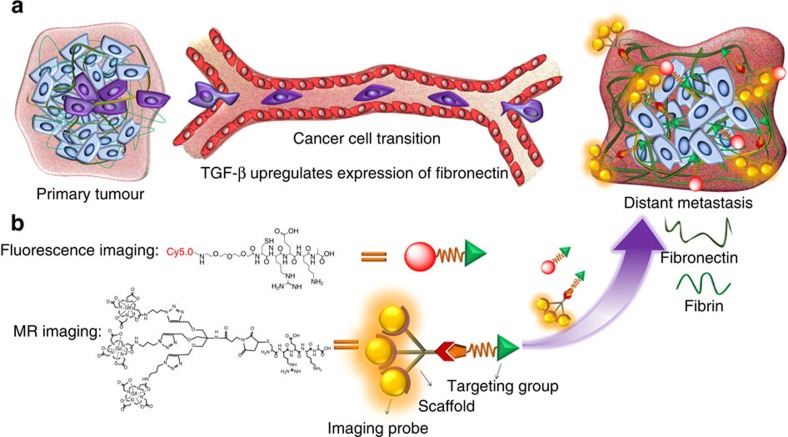
Targeting fibrin–fibronectin complexes for molecular MRI of breast cancer micrometastases. (**a**) Cancer cells from the primary tumour invade into distant organs through epithelial-to-mesenchymal transition (EMT) and transmit signals to prepare ‘soil' of ‘pre-metastatic niche' for metastases. The expression of fibronectin and its associated complexes, such as the fibrin–fibronectin complex, is upregulated by TGF-β. (**b**) The abundant fibrin–fibronectin complexes in the tumour ECM allow the binding of enough CREKA-Tris(Gd-DOTA)_3_ to the ECM marker so as to generate sufficient signal enhancement for effective molecular MRI of small high-risk breast cancer and micrometastases.

**Figure 2:**
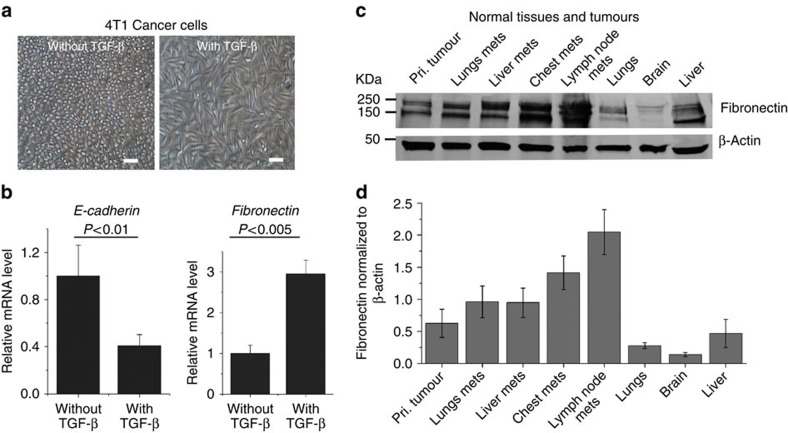
Expression of fibronectin in cells, tissues and tumours. (**a**) The morphology of 4T1 cells with and without TGF-β induction (15 ng ml^−1^, 5 days). Images were taken by phase-contrast microscopy; all scale bars, 50 μm. (**b**) RT–PCR analysis demonstrates that treatment with TGF-β induces upregulation of fibronectin and downregulation of E-cadherin, which are characteristic features of EMT, data represent the mean±s.d., *n*=3. (**c**) Representative western blots showing fibronectin expression in normal tissues, and in primary and metastatic 4T1 breast tumours in Balb/c mice. (**d**) Densitometric analysis of the expression of fibronectin normalized to that of β-actin. Values represent mean±s.d., *n*=3.

**Figure 3:**
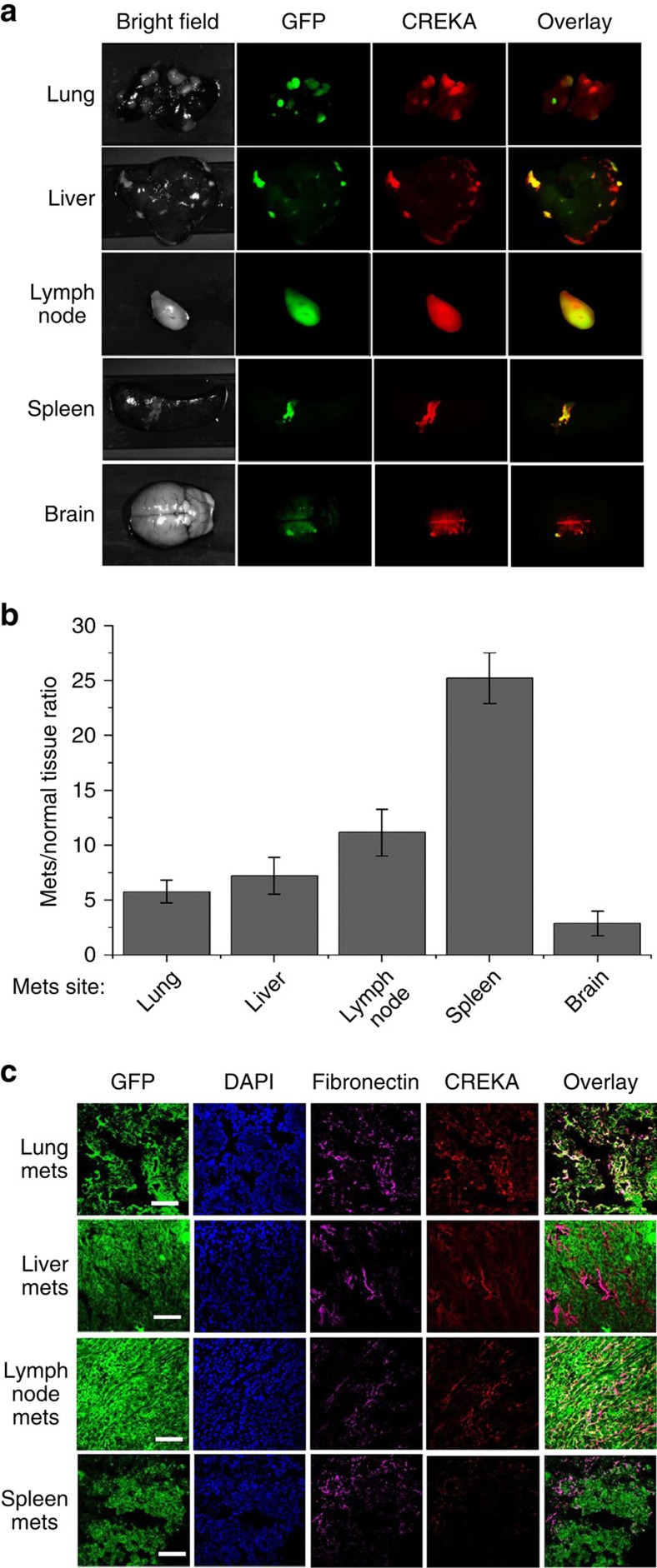
CREKA peptide specifically binds to fibronectin-related complexes in the metastatic tumours. (**a**) Mice bearing spontaneous metastatic 4T1–GFP-Luc2 breast tumours developed by orthotopic implantation were i.v. injected with CREKA-Cy5.0 (0.3 μmol kg^−1^). After 4 h, the mice were killed and tissues with metastases were imaged using the Maestro FLEX *In Vivo* Imaging System. (**b**) Fluorescence intensity ratios between metastatic tumours and normal tissues (*T*/*N* ratio), collected from different mice, represent the mean±s.d., *n*=5. (**c**) Frozen sections of metastatic tumours in the different organs shown in **a** were stained for fibronectin (scale bar, 100 μm).

The binding specificity of CREKA to the fibrin–fibronectin complexes in metastatic tumours was determined in the mouse tumour model using the fluorescence probe CREKA-Cy5.0 (Fig. 1b). Figure 3a and Supplementary Fig. 2 depict the bright-field and fluorescence images of the major organs and tissues with metastases in the mice, 4 h post injection of CREKA-Cy5.0. Tumour metastases labelled with green fluorescent protein (GFP) appear green in various organs and tissues, including brain, liver, lung, lymph node and spleen. Strong red fluorescence from CREKA-Cy5.0 was observed in the metastases, while the normal tissues showed little fluorescence (Fig. 3a,b). The overlay of GFP and Cy5.0 fluorescence images demonstrated specific binding of the targeting peptide to the metastases. In contrast, the non-targeted probe CERAK(Cys-Glu-Arg-Ala-Lys)-Cy5.0 was unable to differentiate between the metastases and normal tissues, as shown in Supplementary Fig. 3.

Immunostaining of the metastatic tumour sections with an antibody against fibronectin showed abundant fibronectin expression in the tumour ECM, as shown in Fig. 3c. The fluorescence from CREKA-Cy5.0 co-localized with the fibronectin immunostaining in the metastatic tumours, while the non-specific peptide probe showed little binding in the tumour sections and no co-localization with the fibronectin staining (Supplementary Fig. 4).

### MRI of metastases in mice with intracardiac cell inoculation

**Figure 4:**
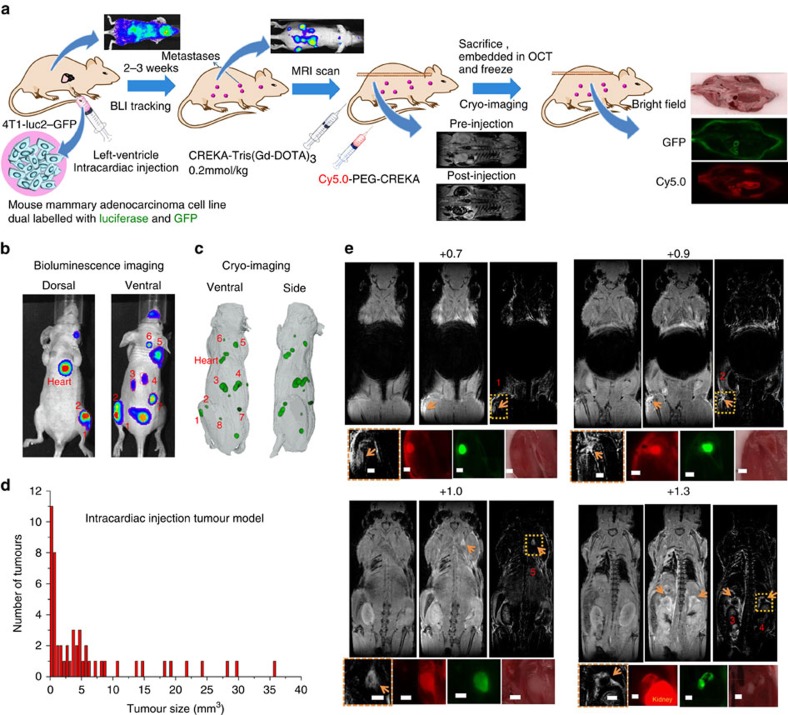
MRI and fluorescence cryo-imaging of metastases in mice with intracardiac-injected 4T1–GFP-Luc2 breast cancer cells. (**a**) Illustration of MRI and fluorescence cryo-imaging of metastases in mice with intracardiac injection of cancer cells. (**b**) Representative bioluminescent images of mice bearing metastases. (**c**) Representative whole-body tumour distribution in mice bearing metastases revealed by cryo-imaging of GFP-labelled tumours and followed by segmentation using Amira software. (**d**) Size distribution of the metastatic tumours after the intracardiac cancer cell inoculation (*n*=4). (**e**) The selected MR images from the 3D data set before and after injection of CREKA-Tris(Gd-DOTA)_3_, and the subtraction images of the pre-injection from the post-injection images, and the enlarged subtraction MR images of metastatic sites and their corresponding cryo-images post-injection of CREKA-Cy5.0 (tumours are indicated by arrow; all scale bars, 1 mm).

Next, we assessed the effectiveness of molecular MRI with CREKA-Tris(Gd-DOTA)_3_ for detecting metastatic tumours in mice bearing 4T1–GFP-Luc2 or MDA-MB-231-Luc breast cancer metastases and further validated it by cryo-fluorescence imaging. The metastatic tumour model was developed by intracardiac inoculation of mice with breast cancer cells (Fig. 4a)^37^,^38^,^39^. Metastatic tumours, including micrometastases, were observed in the lung, liver, lymph node, adrenal gland, bone and brain, using bioluminescence imaging 2 weeks after inoculation. This was also confirmed by fluorescence cryo-imaging^40^ that has the ability to detect single fluorescence-labelled cancer cells (Fig. 4b-d). High-resolution 3D fluorescence images showing the GFP-labelled tumours and Cy5.0-labelled peptide binding in whole mice were acquired and constructed with fluorescence cryo-imaging after contrast-enhanced MRI (Fig. 4c). The tumour detection with cryo-imaging was found to correlate well with the bioluminescence imaging; the former is also more sensitive at detecting very small tumours. Figure 4d shows the number and size distribution of metastatic tumours detected by cryo-imaging. Figure 4e shows representative fat-suppressed T_1_-weighted FLASH MR images of the mice before and after injection of the contrast agent and correlation of tumour enhancement with fluorescence imaging. The targeted contrast agents provided robust contrast enhancement in metastatic tumours, which were clearly visible in MR images. As shown in Fig. 4e, the MRI-detected metastases are located in the bone marrow, adrenal gland and lymph nodes in the thigh and shoulder. The metastases detected by molecular MRI correlated well with those visualized by bioluminescence imaging and post-mortem high-resolution cryo-fluorescence imaging of GFP and CREKA-Cy5.0, which validates the effectiveness of molecular MRI in the detection of small metastatic tumours using the targeted contrast agent. Supplementary Fig. 5 demonstrates examples of MR images enhanced by a non-targeted contrast agent in the same tumour model, indicating that the non-targeted contrast agent is unable to provide significant contrast enhancement to detect small metastatic tumours. The targeted contrast agent CREKA-Tris(Gd-DOTA)_3_ also provided robust contrast enhancement in small metastatic tumours derived from MDA-MB-231 human triple negative breast cancer cells (Supplementary Fig. 6).

**Figure 5:**
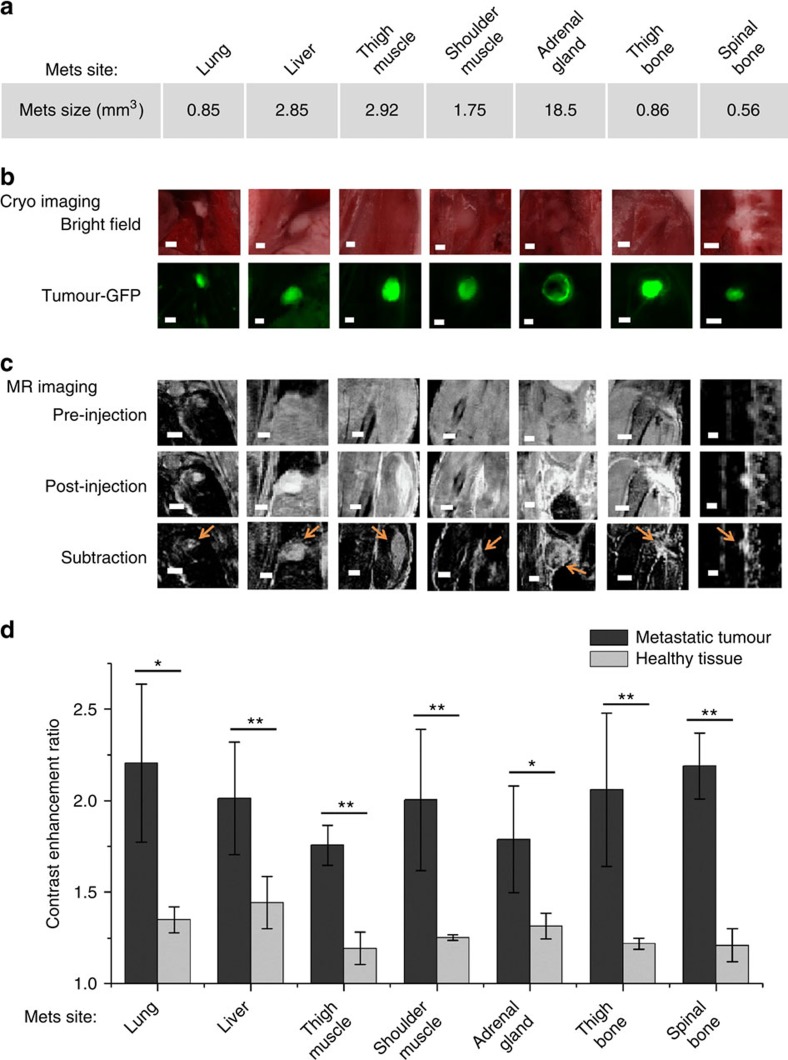
Analysis of tumour metastases detected by MRI and cryo-imaging. The (**a**) size of the given metastatic tumours in different tissues from mice tumour model of intracardiac injection, (**b**) their representative cryo-images, and (**c**) pre-injection MRI, 25 min post-injection MRI and subtraction images of the pre-injection and post-injection MRI are shown (tumours are indicated by arrows; all scale bars, 1 mm). (**d**) The contrast enhancement ratios of metastases in different tissues, *r*=Signal_post-injection_/Signal_pre-injection_. The mean±s.d. were calculated from the tumours (*n*=3–6) in different mice imaged by CREKA-Tris(DOTA)_3_-enhanced MRI (*P<0.05, **P<0.01, Student's *t*-test).

Additional comprehensive analysis of the contrast-enhanced MR images in correlation with fluorescence cryo-imaging revealed robust tumour signal enhancement by CREKA-Tris(Gd-DOTA)_3_ for distinct visualization of small metastatic tumours and micrometastases (0.53–19.50 mm^3^ in volume) in the lung, liver, muscle, bone marrow, lymph nodes and adrenal gland (Fig. 5a–c and Supplementary Fig. 7). The targeted contrast agent resulted in 76–122% signal increase in these metastases, while only 20–48% increase in the background noise was seen in the normal tissues (*P*<0.05) (Fig. 5d). The signal enhancement in the metastases was significantly higher than the increase in background noise, rendering clear tumour delineation, including micrometastases, in the MR images. In contrast, no significant contrast enhancement was observed in metastases in mice injected with the non-targeted contrast agent, CERAK-Tris(Gd-DOTA)_3_ (Supplementary Fig. 5 and Supplementary Fig. 8). The signal increase in metastases in the mice injected with the non-targeted contrast agent was only 31–41%, which is not significantly different from the noise increase in the normal tissues (22–38%, *P*>0.05) (Supplementary Fig. 8).

### Molecular MRI in mice with spontaneous metastasis

**Figure 6:**
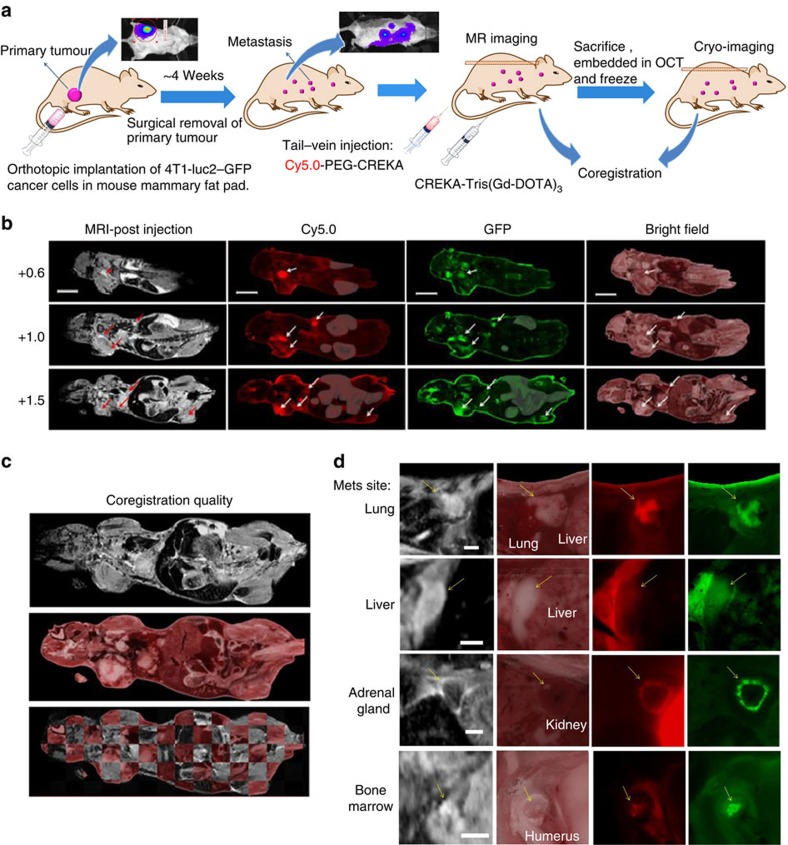
MRI of spontaneous metastases in mice with orthotopic implant of 4T1–GFP-Luc2 breast cancer cells. (**a**) MRI and fluorescence cryo-imaging of cancer metastasis in mouse bearing metastasis, 40 days after cell inoculation in the mammary fat pad. (**b**) Representative sections showing whole-body co-registration of MRI and fluorescence cryo-images (arrows indicate metastases, auto-fluorescence from intestine and stomach is masked; Scale bars, 10 mm). (**c**) The co-registration quality of MRI and cryo-images. (**d**) Representative co-registered MRI (post-injected with the targeted contrast agent) and cryo-images of micrometastases in different organs or tissues (Scale bars, 1 mm).

We next evaluated the ability of molecular MRI with CREKA-Tris(Gd-DOTA)_3_ in imaging micrometastases spontaneously developed in mice bearing orthotopic 4T1–GFP-Luc2 breast cancer xenografts in the mammary fat pad in co-registration with high-resolution fluorescence cryo-imaging (Fig. 6a). This metastatic tumour model closely mimics the physiological symptoms of naturally developed breast cancer metastases^2^,^37^,^38^. Metastatic tumours formed in the mice after the primary tumors were surgically excised^39^. High-resolution 3D MR images, which were contrast-enhanced by CREKA-Tris(Gd-DOTA)_3_, were acquired and co-registered with fluorescence cryo-images of both GFP and CREKA-Cy5.0. Representative co-registration of the contrast-enhanced MR images and cryo-images of bright-field and fluorescent channels are shown in Fig. 6b. A high quality of co-registration was achieved, because there was <100 μm difference between the MRI and cryo-images (Fig. 6c). The targeted contrast agent provided strong contrast enhancement in the metastases (Fig. 6b). Most of the micrometastases labelled with GFP were detected by MRI with CREKA-Tris(Gd-DOTA)_3_. Fluorescence cryo-imaging had the resolution and sensitivity to detect a single GFP-labelled metastatic cancer cell and was able to detect all micrometastases expressing GFP, while molecular MRI was limited by its resolution in detecting the smallest metastases. These metastases were also recognized by CREKA-Cy5.0, as shown in red in the cryo-images, further validating the effectiveness of molecular MRI. Figure 6d shows contrast-enhanced MR images of micrometastases validated by the whole-body image co-registration with both GFP and Cy5.0 fluorescence cryo-imaging. CREKA-Tris(Gd-DOTA)_3_ was able to produce robust contrast enhancement in the MR images of micrometastases as small as 0.125 mm^3^, which enabled the detection of micrometastases in the lung, liver, bone marrow, lymph nodes and other distant organs (Fig. 6d).

### Sensitivity of MRI in detecting cancer micrometastasis

**Figure 7:**
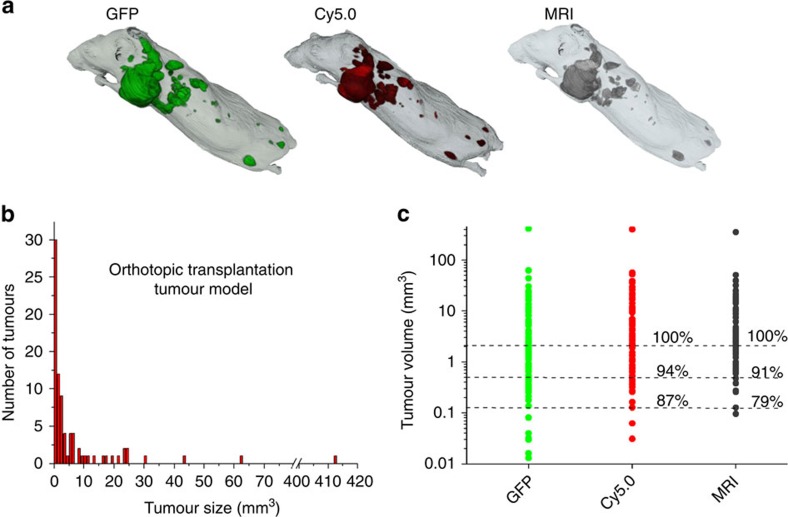
Comparison between the sensitivity of molecular MRI and fluorescent imaging in detecting micrometastases. (**a**) Whole-body distribution of metastatic tumours (GFP), and Cy5.0 (CREKA-Cy5.0) binding or MRI tumour enhancement by CREKA-Tris(DOTA-Gd)_3_ (positive) tumours after segmentation. (**b**) Size distribution of metastatic tumours in mice after orthotopic cancer cell inoculation. (**c**) Sensitivity of molecular MRI in detecting micrometastases, based on volumes of 82 metastatic tumours analysed.

In addition, we also assessed the sensitivity of molecular MRI with CREKA-Tris(Gd-DOTA)_3_ in detecting metastatic tumours, in comparison with fluorescence cryo-imaging. To precisely segment the GFP-labelled tumours, healthy mice were used as controls and bright-field images were also examined to differentiate between tissues with auto-fluorescence and real metastases. Figure 7a shows the metastatic tumours detected with GFP (green) and Cy5.0 (red) fluorescence cryo-imaging and high-resolution contrast-enhanced MRI in a mouse. The tumour size distribution of spontaneous metastases based on GFP fluorescence imaging is shown in Fig. 7b. To quantify the sensitivity of the probes, contrast-to-noise ratio (CNR) was used to ascertain whether a tumour was detected or not. Here the CNR decision threshold was set to 4, which is the most commonly used value^41^,^42^. Figure 7c shows the size and number of metastases in one mouse, detected by different imaging modalities. MRI with CREKA-Tris(Gd-DOTA)_3_ was able to detect 91% of the GFP-labelled metastases with size >0.5 mm^3^, in comparison to 94% in fluorescence imaging with CREKA-Cy5.0. When the minimally detectable tumour size was set to 0.125 mm^3^, molecular MRI had a sensitivity of 79% (Fig. 7c). Fluorescence imaging with CREKA-Cy5.0 was slightly more sensitive than molecular MRI in detecting micrometastases, because a considerably lower concentration of the fluorescence probe is needed to generate detectable signal than molecular MRI. Nevertheless, a sufficient amount of CREKA-Tris(Gd-DOTA)_3_ was able to bind to the abundant fibrin–fibronectin complexes in the tumour ECM to generate significant signal enhancement in detecting micrometastasis with a volume >0.5 mm^3^ by MRI with high sensitivity comparable to fluorescence imaging. We also tested contrast enhancement of a non-targeted contrast agent CERAK-Tris(Gd-DOTA)_3_ along with a non-specific fluorescence probe CERAK-Cy5.0 in the same spontaneous mouse model. MRI with this non-targeted agent was able to detect only large metastases.

To further evaluate tumor detection with molecular MRI, we performed free-response receiver operating characteristic (FROC) studies where the reader of the MR images was blinded to cryo-imaging results. FROC has been used in a variety of medical imaging studies to quantitatively evaluate image quality for tumour detection. Readers identify an arbitrary number of abnormalities per examination and provide the location and confidence level for each perceived abnormality^43^. Data are analysed by comparing with gold standard cryo-imaging. A preliminary FROC study was performed on a 4T1–GFP-Luc2 intracardiac-injected metastatic tumour model. As shown in Supplementary Fig. 9, the FROC curve indicated that the observer could blindly identify 83% metastatic tumours at a rate of 0.22 false positives per examination. In general, undetected tumours had a lower CNR and smaller size than the detected tumours.

### Histological analysis in correlation with molecular MRI

**Figure 8:**
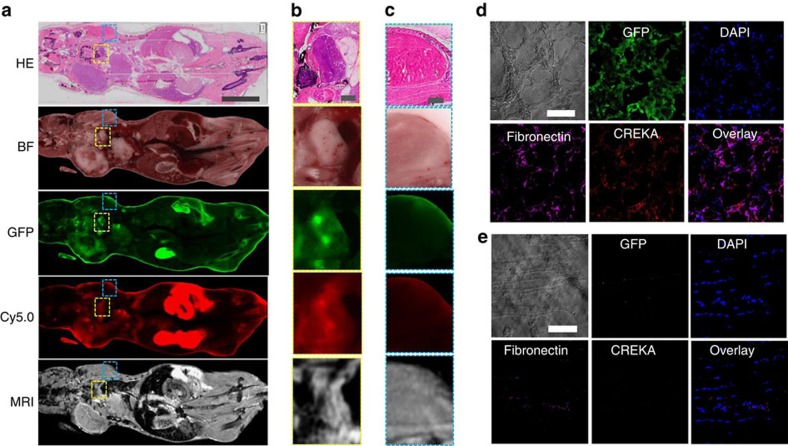
Histological analysis of whole-body cryo-images correlated with MRI. (**a**) Representative whole-body sections of haematoxylin and eosin staining, cryo-images and MR images of a metastasis-bearing mouse injected with CREKA-Cy5.0 and CREKA-Tris(Gd-DOTA)_3_ (Scale bar, 10 mm). Enlarged views of (**b**) a metastatic tumour in the lymph node (indicated by yellow rectangles in **a**) and (**c**) healthy muscle tissue (indicated by blue rectangles in **a**) (Scale bars, 1 mm). The (**d**) selected tumour and (**e**) healthy muscle areas were immunostained with an antibody against fibronectin (Scale bars, 100 μm).

Finally, we performed histological analysis of the cryo-imaged mouse sections to further verify specific binding of the molecular probes in metastatic tumours. Haematoxylin and eosin (H&E) staining of the whole-body sections from cryo-imaging also demonstrated the presence of metastatic tumours in correlation with GFP fluorescence imaging and molecular MRI. Figure 8 shows an example of multimodal examination of a mouse section with metastases. H&E staining revealed the high cell density in the metastatic tumours, which correlated with the strong fluorescence signal and MRI contrast enhancement, in comparison to normal tissues (Fig. 8b,c). Immunostaining further validated the abundant presence of fibronectin in the tumour ECM (Fig. 8d). Co-localization of fibronectin immunostaining with CREKA-Cy5.0 verified the binding of the peptide to fibrin–fibronectin complexes. By comparison, healthy tissues had low contrast enhancement in the post-injection MR images, low Cy5.0 fluorescence signal (Fig. 8a,c) and little fibronectin immunostaining (Fig. 8e).

## Discussion

Contrast-enhanced MRI with small molecular Gd(III) chelates generally demonstrates low sensitivity of molecular imaging of cancer-related cell surface biomarkers. Unlike the biomarkers expressed on the cancer cell surface^44^,^45^, fibronectin and its complexes with other ECM proteins are highly expressed in high-risk breast cancer and distant metastases as compared with normal tissues. In this study, we have demonstrated that abundant fibrin–fibronectin complexes in the ECM of micrometastatic tumours facilitate the binding of a sufficient amount of a small molecular, targeted MRI contrast agent, CREKA-Tris(Gd-DOTA)_3_, to generate robust enhancement for effective molecular MRI of micrometastasis^11^,^46^. By targeting the cancer-associated ECM, we were able to effectively image metastases, including micrometastases, distributed in different distant organs such as the lung, liver, lymph node, adrenal gland and bone. The effectiveness of molecular MRI with CREKA-Tris(Gd-DOTA)_3_ in detecting breast cancer micrometastases was validated by high-resolution fluorescence cryo-imaging of GFP-labelled 4T1 breast cancer cells and the binding of a CREKA-targeted fluorescence probe. Fluorescence cryo-imaging has the ability to detect single GFP-labelled cancer cells^40^, and is a new gold standard in validating the effectiveness and sensitivity of imaging probes and contrast agents for cancer molecular imaging in preclinical development. Three-dimensional, whole-body co-registration of high-resolution MRI images and fluorescence cryo-images in mice enabled us to determine the effectiveness and sensitivity of molecular MRI in detecting micrometastases.

The effectiveness of molecular MRI with CREKA-Tris(Gd-DOTA)_3_ was demonstrated in two different breast cancer metastasis models with both mouse and human carcinoma cells, including intracardiac implant and orthotopic spontaneous models. The former tumour model is relatively convenient to develop reproducible metastases with relatively controlled sizes^47^,^48^. The latter model encompasses all aspects of the metastatic cascade initiated from a primary tumour, which closely resembles clinically relevant metastasis, including the biochemical composition of the tumour ECM. Increased expression of fibronectin and its complexes with fibrin was demonstrated using binding of CREKA-Cy5.0 in both the tumour models, in comparison to the non-binding observed in healthy tissues (Fig. 3). CREKA-Tris(Gd-DOTA)_3_ was able to produce strong signal enhancement to delineate metastatic tumours in both the tumour models during molecular MRI with high spatial resolution, which correlated well with both bioluminescence imaging and fluorescence cryo-imaging.

Currently, X-ray mammography, [18]F-2-deoxy-D-glucose (FDG)-positron emission tomography–computed tomography, ultrasound and contrast-enhanced MRI are commonly used in the detection and clinical management of breast cancer. However, these imaging techniques are not cancer specific and fail to detect micrometastases. Molecular MRI with CREKA-Tris(Gd-DOTA)_3_ shows high specificity and sensitivity in detecting micrometastasis in the mouse tumour models when compared with fluorescence cryo-imaging with CREKA-Cy5.0 in correlation with fluorescence cryo-imaging of GFP-labelled cancer cells. This technique has shown comparable sensitivity (91%) to fluorescence imaging in detecting breast cancer micrometastases with volumes as small as 0.5 mm^3^, with a potential to detect even smaller micrometastases (Fig. 7). For example, bone is the most common site (90%) of breast cancer metastasis and also the most frequent site of relapse after treatment for primary breast cancer^49^. Molecular MRI with CREKA-Tris(Gd-DOTA)_3_ was able to detect bone micrometastasis with a diameter <0.5 mm, as shown in Fig. 6d, demonstrating that this technique has the potential to address the limitations of the current clinical imaging modalities for non-invasive, high resolution and specific detection of micrometastases and other high-risk cancer in the whole body. The goal of cancer molecular imaging is to provide physicians a tool for early detection and differential diagnosis of high-risk tumours with confidence. The preliminary FROC blind analysis has shown 83% overall sensitivity in detecting metastatic tumours by molecular MRI with CREKA-Tris(Gd-DOTA)_3_. The FROC blind analysis had no apparent criteria and the results might be affected by the experience of the reader. Further comprehensive work is needed to optimize the imaging protocols and to establish the imaging analysis criteria with a larger sample size for accurate early detection and diagnosis of very small aggressive tumours with molecular MRI.

Numerous clinical studies have shown that increased expression of fibronectin is associated with high-risk breast cancer with poor prognosis. For example, an immunohistochemical examination of tumour specimens from 110 breast cancer patients revealed that tumours with high fibronectin expression were significantly associated with a higher probability of metastasis and poorer overall survival^50^. Since the high expression of fibronectin is a marker of EMT and is associated with high-risk breast cancer, molecular MRI with CREKA-Tris(Gd-DOTA)_3_ has the potential to non-invasively grade and differentiate the aggressiveness of small breast cancer and to assess tumour heterogeneity, which forms a major challenge in the clinical management of breast cancer. Molecular MRI with CREKA-Tris(Gd-DOTA)_3_ also can be used in non-invasive assessment of the efficacy of therapeutics in treating metastatic breast cancer^51^.

CREKA-Tris(Gd-DOTA)_3_ is a small molecular MRI contrast agent, with a molecular weight of 2,913 Da. The small size allows rapid clearance of the unbound Gd(III)-based contrast agent from the body via renal filtration, which is an advantageous safety feature for clinical development of the contrast agent. Delayed excretion and tissue accumulation of any Gd(III)-based contrast agent may result in potential unintended toxic side effects, such as nephrogenic systemic fibrosis (NSF)^52^. We previously demonstrated that CREKA-Tris(Gd-DOTA)_3_ is readily cleared from the body, resulting in low tissue retention^28^. Rapid and complete clearance of the targeted Gd(III)-based contrast agent is essential to minimize any contrast-associated toxicity, and can also minimize background signal for tumour-specific contrast-enhanced MRI. Thus, molecular MRI with CREKA-Tris(Gd-DOTA)_3_ has the potential to be developed for clinical management of breast cancer.

In summary, we demonstrate, for the first time, the effectiveness of molecular MRI with a small molecular peptide-targeted MRI contrast agent in the detection of breast cancer micrometastases. Early detection and accurate diagnostic imaging of high-risk breast cancer, including micrometastases, play an important role in the clinical management of breast cancer and in tailoring efficacious treatments, with the goal of improving patient outcomes. Molecular MRI with CREKA-Tris(Gd-DOTA)_3_ can potentially open new avenues for the clinical management of breast cancer and to treat micrometastases at an early stage. The molecular MRI technique can also be used for breast cancer screening in high-risk patient populations, and for monitoring disease progression and therapeutic response. Thus, molecular MRI with the small molecular peptide-targeted contrast agent holds great promise for clinical MR cancer molecular imaging.

## Methods

### Materials and animals

The imaging probes and contrast agents, CREKA-Cy5.0, CERAK-Cy5.0, CREKA-Tris(Gd-DOTA)_3_, and CERAK-Tris(Gd-DOTA)_3_ were synthesized as previously described^28^. D-Luciferin (Gold Biotechnology, St Louis, MO), TGF-β (Abcam, Cambridge, MA), RNeasy Plus Kit (Qiagen,Valencia, CA), High Capacity cDNA Transcription Kit (Applied Biosystems, Waltham, MA), Mastercycler ep realplex2 (VWR, Radnor, PA), homogenization buffer, protease inhibitor cocktail (Sigma-Aldrich; P8340), SDS–PAGE gels, polyvinylidene difluoride membranes (Bio-Rad Laboratories, Hercules, CA), anti-fibronectin polyclonal antibody (Abcam; 1:4000), anti-β-actin antibody (Cell Signaling Technology, Danvers, MA; 1:1000), rhodamine-red-X-conjugated goat anti-rabbit IgG (H+L) (Jackson ImmunoResearch Laboratories, West Grove, PA; 1:200) and other related reagents were purchased and used according to the manufacturers' instructions. The 4T1–GFP-Luc2 (metastatic murine mammary carcinoma, catalogue number: 128090) and MDA-MB-231-Luc (metastatic human breast carcinoma, catalogue number:124319) breast cancer cell lines were purchased from Caliper Life Sciences (Hopkinton, MA) and have been authenticated. Cells were maintained as recommended by the provider. All the mice were obtained from Charles River and housed in the Animal Core Facility at Case Western Reserve University. All animal experiments were performed in accordance with the animal protocol approved by the CWRU Institutional Animal Care and Use Committee.

### Mammary fat pad spontaneous metastasis model

Seven- to 8-week-old female BALB/c mice were anaesthetized and 4T1–GFP-Luc2 breast cancer cells (5 × 10^5^ in 40 μl PBS) were injected into the mammary fat pad for tumour induction. To monitor tumour growth and metastases, the mice were i.p. injected with D-luciferin (200 μl in PBS, 15 mg ml^−1^). After 10 min, the mice were anaesthetized by 2% isoflurane and imaged using the Xenogen IVIS Lumina system (Caliper Life Sciences). In some experiments, the lower portion of each animal was shielded before reimaging to minimize the bioluminescence from the primary tumour and observe the signals from the metastatic regions. To control morbidity associated with excess primary tumour burden, the primary tumour was surgically removed after 4 weeks, once metastasis was evident during bioluminescent imaging. The mice were maintained for 10 additional days and killed for further study.

### Intracardiac-injected metastasis model

Seven- to 8-week-old female BALB/c nude mice were anaesthetized with 2–3% isoflurane in O_2_ and injected in the left ventricle of the heart with 1 × 10^5^ 4T1–GFP-Luc2 (metastatic murine mammary carcinoma) or 1 × 10^5^ MDA-MB-231-Luc (metastatic human breast carcinoma) breast cancer cells and 0.5 mg of D-luciferin mixed in 100 μl PBS. The mice were immediately imaged by bioluminescence imaging (BLI) to confirm for successful injections into the left ventricle. A successful intracardiac injection is indicated by images showing systemic distribution of bioluminescence in the animal. Only mice with evidence of a successful injection were used in the experiment. The subsequent metastasis was monitored *in vivo* by BLI. Mice bearing 4T1–GFP-Luc2 or MDA-MB-231-Luc metastatic tumours were studied at 2–3 weeks post injection.

### qPCR analysis of cancer cells

4T1 cancer cells were seeded onto a 6-well plate at a density of 5,000 cells per well and cultured with TGF-β (15 ng ml^−1^) for 5 days at 37 °C and 5% CO_2_. The control groups were seeded and incubated in identical conditions without the addition of TGF-β. Total RNA was collected from the cell samples, isolated using an RNeasy Plus Kit, and then reverse-transcribed into complementary DNA (cDNA) using the High Capacity cDNA Transcription Kit. Semiquantitative real-time PCR was carried out using a SYBR Green Master Mix (Life Technologies, Carlsbad, CA), according to the manufacturer's recommendations. The gene expression of the individual genes examined was normalized to the corresponding GAPDH signals. Both cDNA synthesis and real-time PCR were carried out on the Mastercycler ep realplex2. The relative mRNA expression levels were calculated using the 2^−ΔΔCT^ method. Real-time PCR primer sequences are as follows: msFN-sense, 5′-CGGTAGGACCTTCTATTCCT-3′; msFN-antisense, 5′-CGGTAGGACCTTCTATTCCT-3′; E-cad-sense, 5′-CATCTTTGTGCCTCCTGAAA-3′; E-cad-antisense, 5′-AGTAGAGGCAGGGATGTT-3′; GAPDH-sense, 5′-TCCATGACAACTTTGGTATTCGT-3′; GAPDH-antisense, 5′-AGTAGAGGCAGGGATGATGTT-3′.

### Western blot

Metastatic tumours and normal tissues (30–100 mg) were homogenized in 200–500 μl of homogenization buffer mixed with the protease inhibitor cocktail. The resulting lysates were centrifuged for 10 min at 10,000*g* at 4 °C. The supernatants were collected, and the protein concentration was determined by protein assay (Bio-Rad). SDS–PAGE and western blotting were performed using 25 μg proteins. The blots were incubated with a rabbit anti-mouse fibronectin polyclonal antibody; the anti-β-actin antibody was used for the loading control. The blots were washed and incubated with Rhodamine-Red-X conjugated goat anti-rabbit IgG (H+L). Typoon scanner was used for processing the membrane blotted with Rhodamine-Red-conjugated secondary antibody. The data were analysed using ImageJ software.

### Tumour metastasis fluorescence imaging

Fluorescence imaging of 4T1–GFP-Luc2 breast cancer metastasis was performed on a Maestro FLEX *In Vivo* Imaging System using the appropriate filters for GFP (excitation: 444–490 nm; emission: 515 nm long-pass filter; acquisition settings: 500–720 in 10-nm steps) and Cy5 (excitation: 576–621 nm; emission: 635 nm long-pass filter; acquisition settings: 630–800 in 10-nm steps). The mice bearing 4T1–GFP-Luc2 metastasis (orthotopic model) were killed 4 h after an i.v. injection of CREKA-Cy5.0 or CERAK-Cy5.0 (10 nmol Cy5.0 per mouse). Both GFP and Cy5.0 fluorescent images of the organ or the tissue with metastasis were acquired. The fluorescent signal was spectrally separated from the multispectral fluorescence images with Maestro software (Cambridge Research & Instrumentation, Inc, Woburn, MA) to subtract the background auto-fluorescence.

### Tumour metastasis immunofluorescence

Metastatic tumours from different organs and tissues of mice injected with Cy5.0-labelled peptides were embedded in optimal cutting temperature compound (O.C.T) and cryo-sectioned into 5-μm slices. For fibronectin immunostaining, the tissues were stained with a rabbit anti-mouse fibronectin polyclonal antibody, followed by Rhodamine-Red-X-conjugated goat anti-rabbit IgG (H+L). The nuclei were stained with DAPI (4′,6-diamidino-2-phenylindole). Tissue slides were imaged on an Olympus FV1000 confocal laser scanning microscope. GFP was observed using 405-nm laser, the emission wavelength was read from 480–495 nm and is expressed as green. DAPI was observed using 405-nm laser, the emission wavelength was read from 450–470 nm and is expressed as blue. Rhodamine-Red was observed using 543-nm laser, the emission wavelength was read from 560–620 nm and is expressed as purple. Cy5 was observed using 635 nm laser, the emission wavelength was read from 655–755 nm and is expressed as red.

### *In vivo* MRI

The MRI study was performed using a Bruker Biospec 7 T MRI scanner (Bruker Corp., Billerica, MA, USA) with a volume radio frequency coil. Mice were anaesthetized with a 2% isoflurane–oxygen mixture in an isoflurane induction chamber. The tail vein was catheterized with a 30 gauge needle connected with 1.6-m tubing filled with heparinized saline. The animal was then moved into the magnet and kept under inhalation anaesthesia with 1.5% isoflurane–oxygen via a nose cone. A respiratory sensor connected to a monitoring system (SA Instruments, Stony Brook, NY) was placed on the abdomen to monitor rate and depth of respiration. The body temperature was maintained at 37 °C by blowing hot air into the magnet through a feedback control system. Images were acquired with a fat suppression 3D T1-weighted FLASH sequence with respiratory gating before injection (Sequence 1: repetition time (TR)=25 ms, echo time (TE)=2.8 ms, average=3, 15° flip angle, in-plane field of view (FOV)=6 cm, 18-mm slab thickness. Resolution: 0.1172 × 0.09766 × 0.562 mm, scan duration with respiratory gating: 20 min). After the pre-injection baseline MR imaging, the targeted contrast agent or the non-targeted scrambled control agent was injected at a dose of 0.2 mmol of Gd per kg by flushing with 80 μl saline. After 5 min, T_1_-weighted FLASH sequence was acquired. For MR and cryo-imaging combined study, mice were pre-settled in a mold and then killed after live imaging by using an i.v. injection of 50 μl ketamine–xylazine. A T_1_-weighted fat suppression 3D FLASH sequence with high resolution was acquired (Sequence 2: TR=80 ms, TE=2.8 ms, average=3, 15° flip angle, FOV=6 cm, 18-mm slab thickness. Resolution: 0.1172 × 0.09766 × 0.1406, mm, scan duration: 2 h 11 min).

### MRI and bioluminescence imaging

Mice with metastasis were i.p. injected with D-luciferin (200 μl in PBS, 15 mg ml^−1^). Ten minutes following the injection, the mice were anaesthetized by 2% isoflurane and imaged using Xenogen IVIS Lumina system. Images of both the dorsal and ventral view were obtained. The mice were imaged by MRI the next day. Images of pre and post injection of contrast agents were acquired by using Sequence 1.

### Cryo-imaging and histological analysis

Mice after MRI with Sequence 2 were held in the mold to maintain their position. The mold was filled with O.C.T to embed the mice and then flash frozen using liquid nitrogen. The frozen mice were sectioned and imaged at 10.472 × 10.472 μm in plane resolution and 50-μm section thickness using CryoViz (Bioinvision Inc, Cleveland, OH). Bright-field, GFP and Cy5.0 images were acquired using a liquid crystal RGB filter and monochrome camera. Fluorescence images of excitation and emission were acquired using dual band FITC/Cy5 fluorescence filters (Exciter: 51008 × , Dichroic: 51008bs, Emitter: 51008, m; Chroma, Rockingham, VT). Typically, bright-field, green and red fluorescence volumes were about 120 GB for each mouse. Histological sections were obtained by the tape transfer method^53^. This method depends on a special adhesive tape called CryoFilm, which was placed on the surface of the samples. When the blockface was sectioned, the tissue section was left attached to the CryoFilm. The tissue sections were then used for H&E staining and immunostaining.

### MR image analysis

The CNR of segmented tumours in the MR images was calculated using the following equation: CNR=(*S*_T_−*S*_N_)/(*σ*_n_), where *S*_T_ and *S*_N_ denote the signal in tumour and its relative normal tissue, respectively, and *σ*_n_ is the s.d. of noise estimated from the background air. The CNR of Cy5.0 fluorescent imaging in the segmented tumour was similarly calculated.

### 3D registration of fluorescence cryo-images to MR images

The bright field of the cryo-image volume was used as the reference volume and the other image data (3D MRI and 2D histology) were registered to it. The grey scale, rigid, affine and non-rigid registration of the moving MRI volume was registered to the reference 3D bright field. Nifty software packages were implemented for designing the algorithms.

### Blind image analysis

The FROC observer-performance experiment was used to perform blind analysis. An observer, experienced in reading mouse MRI images, blindly evaluated all MRI images. The observer was aware of the diagnosis of metastatic cancer but was unaware of cryo-imaging data. The entire MRI image volume was blindly evaluated by the observer. The observer was asked to indicate the location of the tumour and give a confidence rating from 1 to 4, with 1 being least possible and 4 being very likely. CNR of the detected tumours and undetected tumours were measured. Selected tumour locations were evaluated based on GFP signal on cryo-images. Relative positions of both GFP volume and observer identified locations were visualized in 3D with Amira software.

## Additional information

**How to cite this article**: Zhou, Z. *et al.* MRI detection of breast cancer micrometastases with a fibronectin-targeting contrast agent. *Nat. Commun.* 6:7984 doi: 10.1038/ncomms8984 (2015).

## Supplementary Material

Supplementary Information Supplementary Figures 1-9
