# Supplementary material for: MRI detection of breast cancer micrometastases with a fibronectin-targeting contrast agent
Source: Nat Commun. 2015 Aug 12;6:7984. doi: 10.1038/ncomms8984 (PMC4557274; doi:10.1038/ncomms8984)
Supplement: Supplementary Information — Supplementary Figures 1-9 [file ncomms8984-s1.pdf]

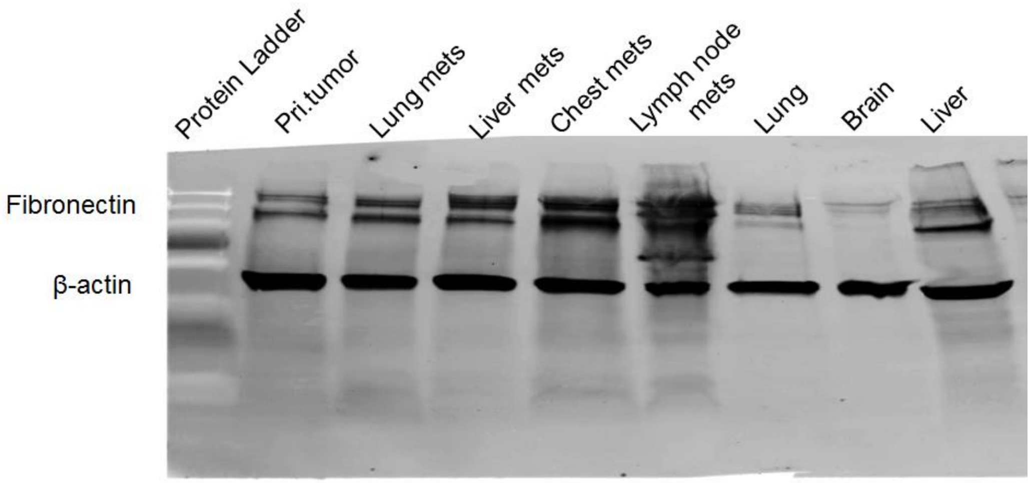

Supplementary Figure 1. Representative western blots with full gel and protein ladder showing fibronectin expression in normal tissues, and in primary and metastatic 4T1 breast tumors in Balb/c mice.

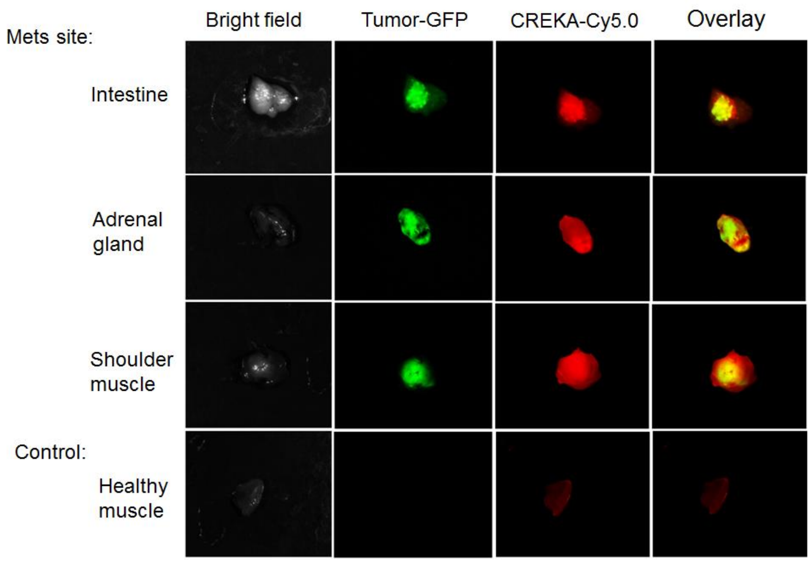

Supplementary Figure 2. Mice bearing 4T1-GFP-Luc2 breast metastatic tumor were intravenously injected with CREKA-Cy5.0 (0.3 μmol/kg body weight). After 4 h, the mice were sacrificed and the tissues with metastases were imaged with the Maestro FLEX In Vivo Imaging System.

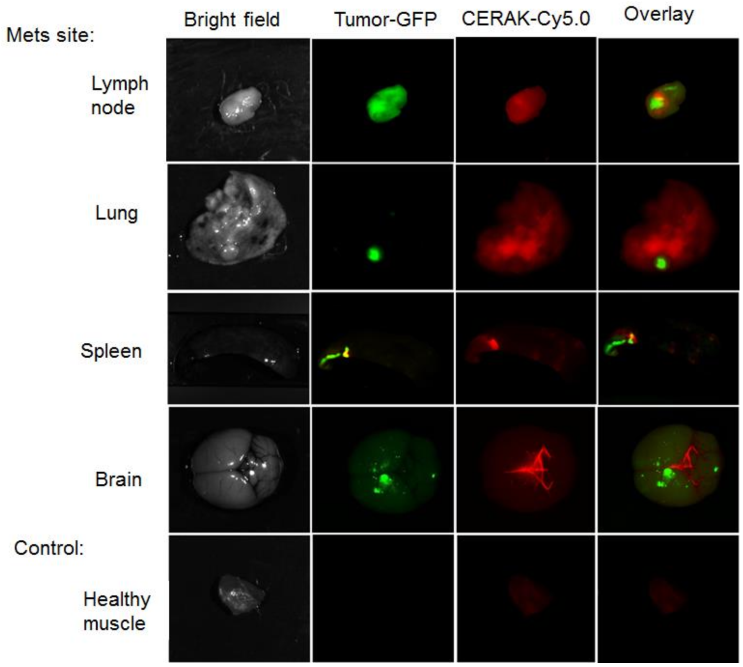

Supplementary Figure 3. Mice bearing 4T1-GFP-Luc2 breast metastatic tumor were intravenously injected with CERAK-Cy5.0 (0.3 μmol/kg body weight). After 4 h, the mice were sacrificed and the tissues with metastases were imaged with the Maestro FLEX In Vivo Imaging System.

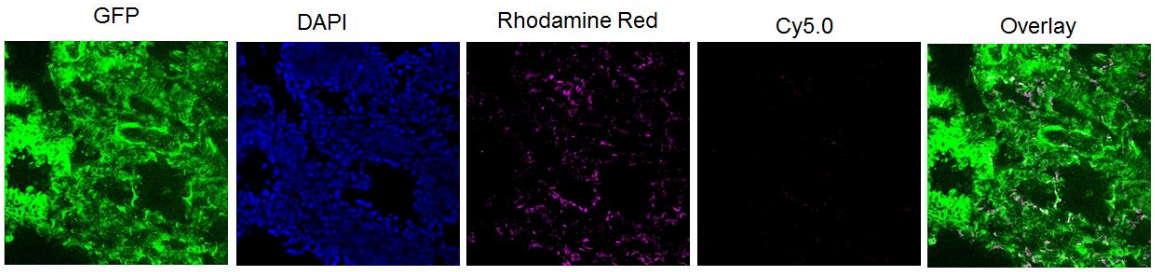

Supplementary Figure 4. Frozen sections of metastatic tumors from lung of mice 4 h post-injected with CERAK-Cy5.0 (0.3 μmol/kg body weight) were stained for fibronectin.

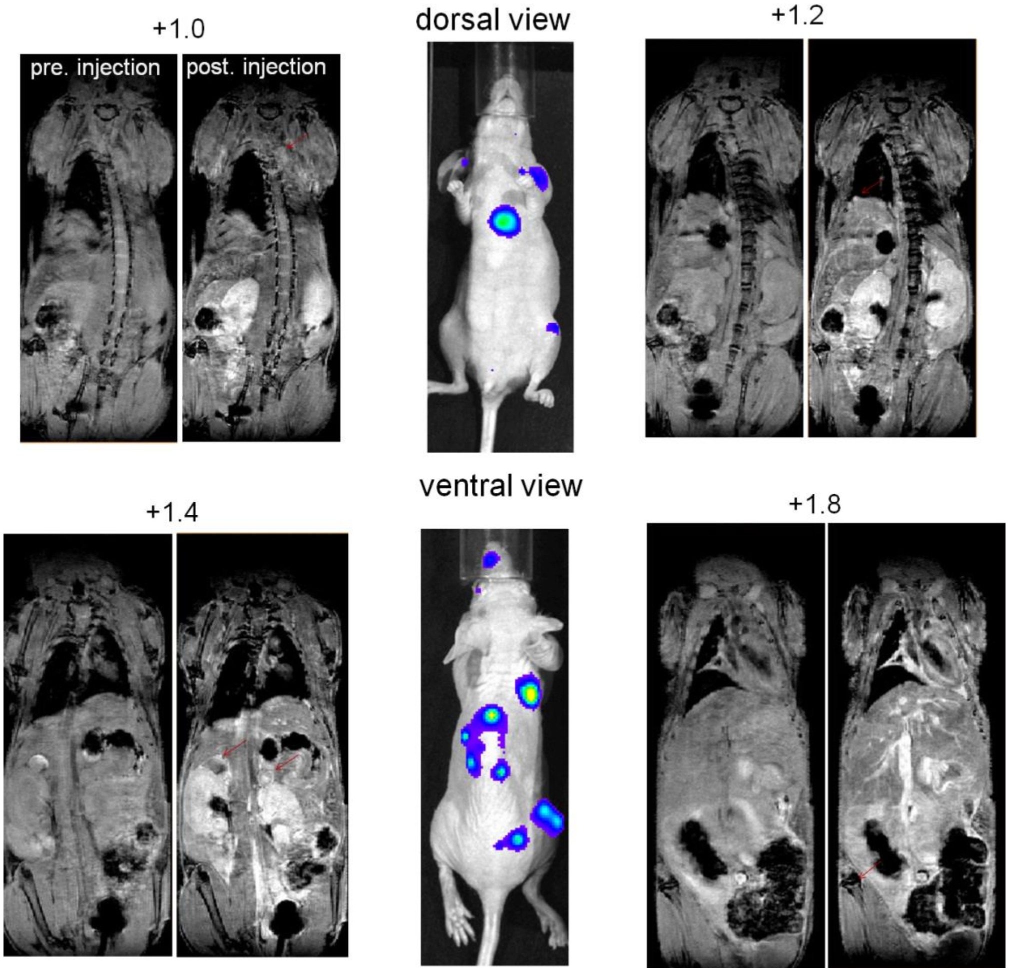

Supplementary Figure 5. Examples of bioluminescence images of mouse with intracardiac-injected 4T1 metastasis and their corresponding MR images from 3D dataset before and post injection of non-targeted contrast agent, CERAK-Tris(Gd-DOTA)$_3$ (red arrow point to metastatic sites).

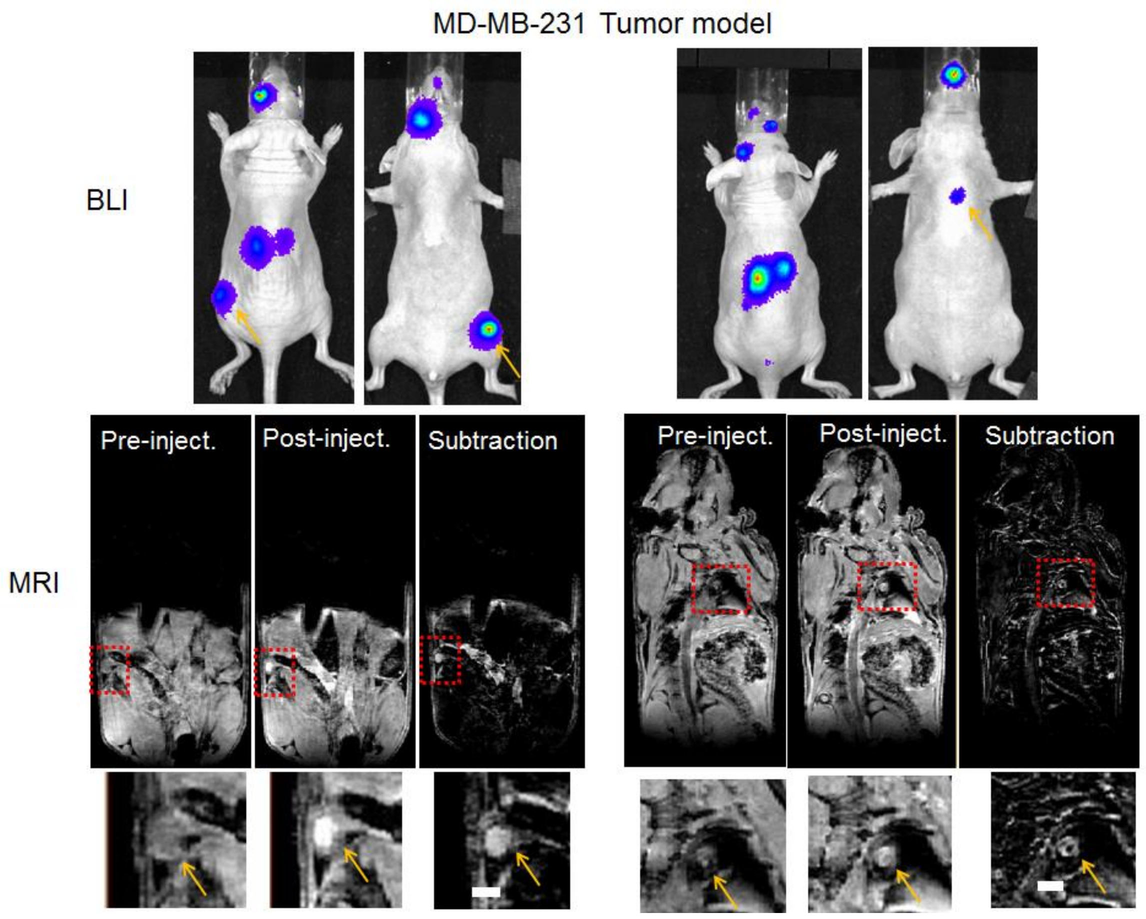

Supplementary Figure 6. Representative MRI and bioluminescent images of metastases in mice with intracardiac injection of MDA-MB-231-Luc breast cancer cells. The selected MR images from the 3D dataset before and after injection of CREKA-Tris(Gd-DOTA)$_3$, and the subtraction images of the pre-injection and post-injection MRI, and the enlarged images of metastatic sites (tumors are indicated by arrow, scale bars are 1 mm). The metastatic tumors were confirmed by cryo-imaging.

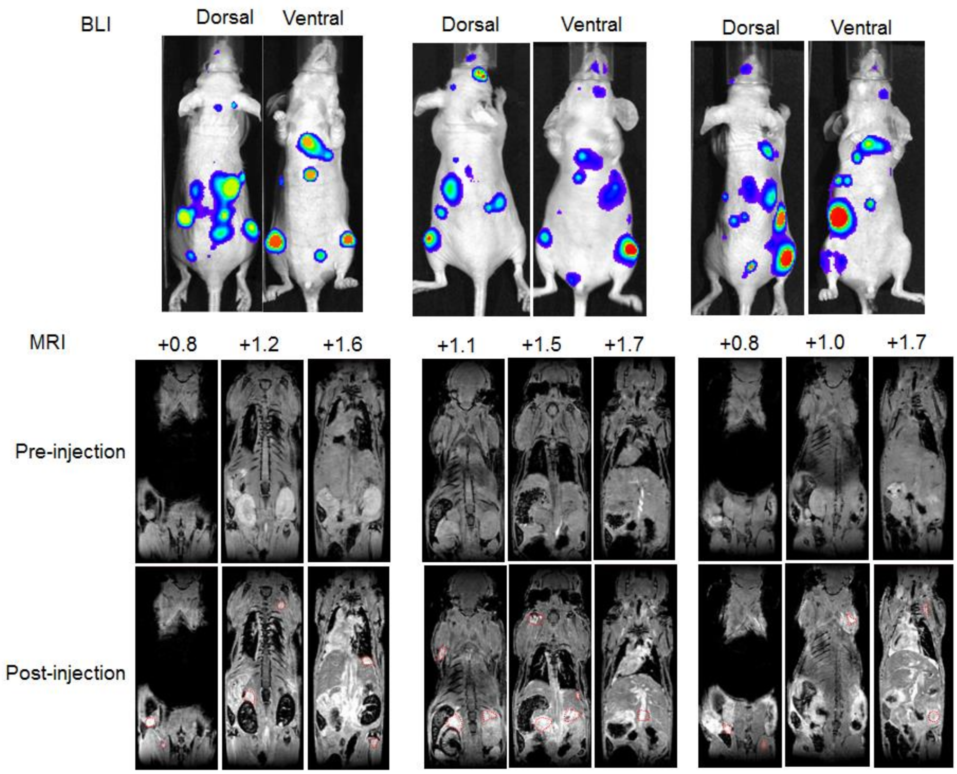

Supplementary Figure 7. Examples of bioluminescence images of mice with intracardiac-injected 4T1 metastasis and their corresponding MR images from 3D dataset before and post injection of CREKA-Tris(Gd-DOTA)$_3$, (Tumors were marked by dotted red lines).

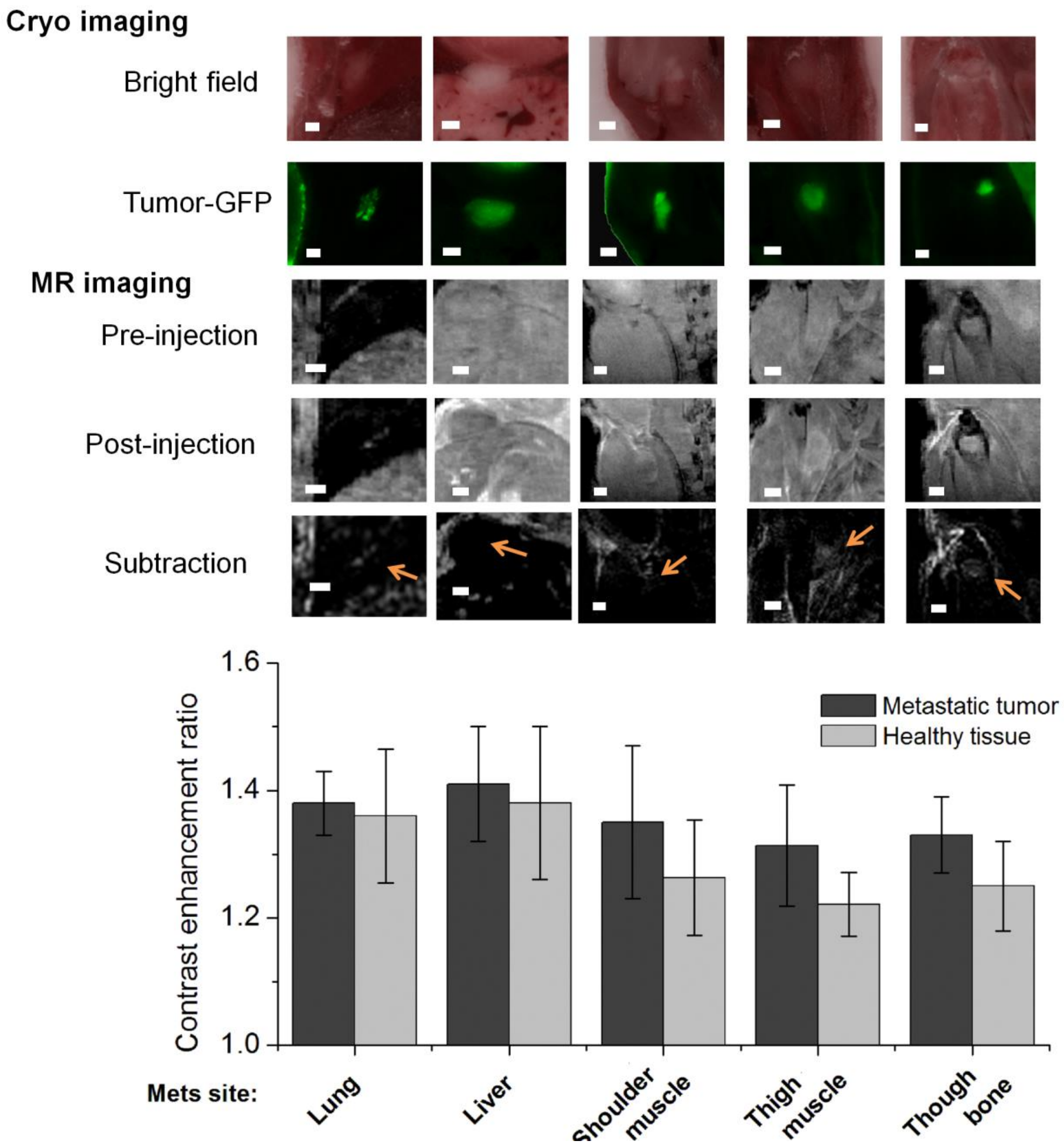

Supplementary Figure 8. Representative MR and cryo-images of metastatic sites from mice pre or post-injected with the nontargeted contrast agents, CERAK-Tris(Gd-DOTA)$_3$. Subtraction images of the pre-injection and post-injection MRI are also shown (tumors are indicated by arrow, all scale bars are 1 mm). The contrast enhancement ratios of metastases and their corresponding normal tissues, r = Signal$_{post\text{-}injection}$/Signal$_{pre\text{-}injection}$, Data represent the mean values ± standard deviation, n = 3–6, data were collected from different mice imaged by CERAK-Tris(DOTA)$_3$ enhanced MRI.

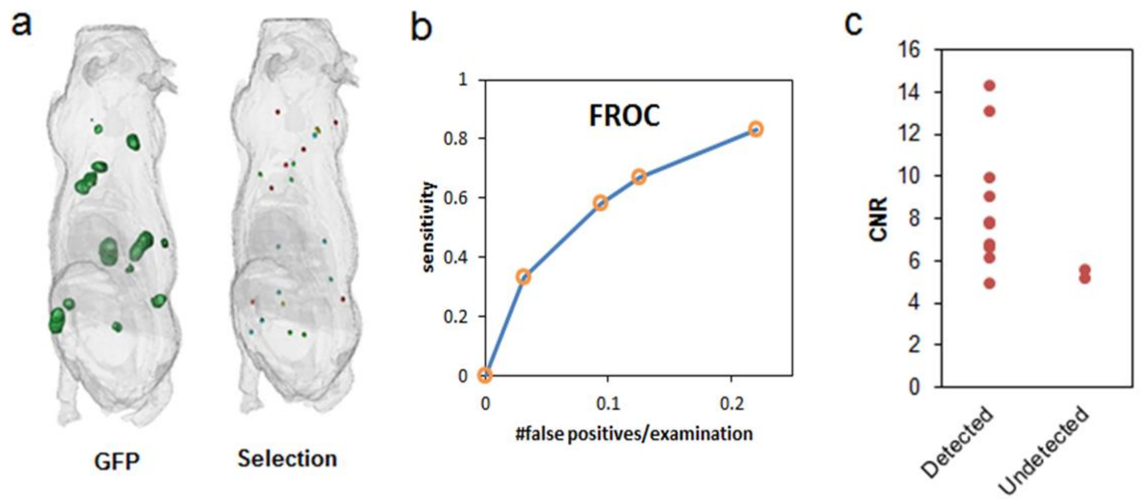

Supplementary Figure 9. Graph shows blind analysis of free-response receiver operating characteristic (FROC) observer-performance curves for CREKA-Tris(Gd-DOTA)$_3$ enhanced molecular MRI of 4T1-GFP-Luc2 metastatic tumors in an intracardiac implantation tumor model. (a) Representative whole-body tumor distribution in mice bearing metastases revealed by cryo-imaging of GFP-labeled tumors (left) and the tumor position blindly selected by the observer (right) with confidence ratings from 1 (least likely) to 4 (most likely). Color scheme is: green: level 1; yellow: level 2; red: level 3; blue: level 4. (b) FROC analysis giving a plot of the sensitivity (TP/[TP+FN]), which is equal to the percentage of detected tumors, as a function of the number of false positives per examination. (c) Contrast to noise ratio of detected and undetected tumors. At an operating point of 0.22 false positives per examination, the sensitivity is 83%.
